# Parasites may help stabilize cooperative relationships

**DOI:** 10.1186/1471-2148-9-124

**Published:** 2009-06-01

**Authors:** Ainslie EF Little, Cameron R Currie

**Affiliations:** 1 Department of Bacteriology, University of Wisconsin-Madison, Madison, WI 53706, USA; 2 Smithsonian Tropical Research Institute, Apartado Box 2072, Balboa, Ancon, Panama; 3 Department of Ecology and Evolutionary Biology, University of Kansas, Lawrence, KS 66045, USA

## Abstract

**Background:**

The persistence of cooperative relationships is an evolutionary paradox; selection should favor those individuals that exploit their partners (cheating), resulting in the breakdown of cooperation over evolutionary time. Our current understanding of the evolutionary stability of mutualisms (cooperation between species) is strongly shaped by the view that they are often maintained by partners having mechanisms to avoid or retaliate against exploitation by cheaters. In contrast, we empirically and theoretically examine how additional symbionts, specifically specialized parasites, potentially influence the stability of bipartite mutualistic associations. In our empirical work we focus on the obligate mutualism between fungus-growing ants and the fungi they cultivate for food. This mutualism is exploited by specialized microfungal parasites (genus *Escovopsis*) that infect the ant's fungal gardens. Using sub-colonies of fungus-growing ants, we investigate the interactions between the fungus garden parasite and cooperative and experimentally-enforced uncooperative ("cheating") pairs of ants and fungi. To further examine if parasites have the potential to help stabilize some mutualisms we conduct Iterative Prisoner's Dilemma (IPD) simulations, a common framework for predicting the outcomes of cooperative/non-cooperative interactions, which incorporate parasitism as an additional factor.

**Results:**

In our empirical work employing sub-colonies of fungus-growing ants, we found that *Escovopsis-*infected sub-colonies composed of cheating populations of ants or fungi lost significantly more garden biomass than sub-colonies subjected to infection or cheating (ants or fungi) alone. Since the loss of fungus garden compromises the fitness of both mutualists, our findings suggest that the potential benefit received by the ants or fungi for cheating is outweighed by the increased concomitant cost of parasitism engendered by non-cooperation (cheating). IPD simulations support our empirical results by confirming that a purely cooperative strategy, which is unsuccessful in the classic IPD model, becomes stable when parasites are included.

**Conclusion:**

Here we suggest, and provide evidence for, parasitism being an external force that has the potential to help stabilize cooperation by aligning the selfish interests of cooperative partners in opposition to a common enemy. Specifically, our empirical results and IPD simulations suggest that when two mutualists share a common enemy selection can favor cooperation over cheating, which may help explain the evolutionary stability of some mutualisms.

## Background

The stability of cooperation is an evolutionary paradox – partners should be selected to cheat, pursuing their own selfish interests by obtaining benefits without providing a reward in return. Despite the inherent selfishness of individuals, cooperation within and between species is common in nature [1-3]. Hamilton's Kin Selection Theory [4] helps explain cooperation among closely related individuals: organisms increase their fitness through altruism with close relatives due to their shared genes. The main theories used to help explain cooperation among unrelated individuals have been categorized as either directed reciprocation or by-product benefits [5]. Many of the models that fit the former category, including host sanction and partner fidelity, have developed out of, and are supported by, years of game theory modelling and focus on how individuals avoid being exploited by their partners [6-10]. Specifically, within directed reciprocation cooperation is thought to be maintained when partners prevent one another from pursuing their own selfish interests ("cheating"), such as retaliation against cheating. However, empirical support for mechanisms of directed reciprocation that stabilize interspecific mutualisms is mostly lacking [5,10].

Studies on the stability of mutualisms have generated extensive and valuable information about cooperation between unrelated individuals. However, these studies have primarily been framed within the traditional view of pair-wise partner associations occurring in isolation [10-12], while it is becoming increasingly clear that mutualisms are usually embedded within complex ecological communities [11,13-22], and that these additional symbionts or interactants (tertiary, quaternary etc.) play important roles in mutualism dynamics. Indeed, third parties have been shown to alter the intensity, outcomes, and ultimately even the symbiotic state (mutualistic or parasitic) of an association (see [11] for review). For example, some mutualisms are known to exist only in the presence of other species, such as protective mutualisms where the presence of natural enemies is required for benefits to be conferred [17,23-26]. In addition, recent work on bird-dispersed pine trees has revealed that the presence/absence of a competitor, pine squirrels, alters selection on a trait specifically associated with the bird-pine mutualism [27]. This illustrates that selection imposed on a mutualism by a third party can disrupt the success and/or stability of the association. Despite mounting evidence to support the importance of additional symbionts and community members in the biology of mutualisms, the concept has not yet been extensively explored with respect to the evolutionary stability of mutualisms.

Parasites of mutualisms may be particularly important in altering the dynamics of cooperative relationships in ways that influence their stability. Parasites not only drive host evolution, they also shape community dynamics by indirectly influencing the organisms their hosts interact with. The indirect influence of parasites should be especially pronounced within mutualistic relationships, as increased morbidity and mortality caused by a parasite of one partner will significantly influence the success of the other partner [28,29]. Since cooperative partners frequently face a 'common enemy' in the form of parasites, we hypothesize that the presence of an abundant and virulent parasite of one member of a mutualism could provide selective pressure such that cooperation between partners is favored over exploitation. Our hypothesis is similar to triadic models developed by social scientists to investigate the role of third parties in cooperative dynamics among humans [30], however as mentioned above, the impact of additional players on the evolutionary stability of mutualisms has not been examined empirically.

The mutualism between fungus-growing ants and the fungi they cultivate for food is an example of a cooperative relationship that has persisted over evolutionary time despite continual impact from a virulent parasite [1,28,31-33]. To test our hypothesis that parasites may stabilize cooperative relationships, we experimentally manipulated sub-colonies of fungus-growing ants to determine the impact of parasites on (i) ants with fungal partners who provide decreased benefit to ants (cheater fungi), and (ii) fungi being tended by ants who provide limited benefits and increased costs to the fungus garden (cheater ants). We explore our empirical results further by utilizing the classic Iterative Prisoner's Dilemma model [34] to confirm how the addition of a virulent parasite influences the traditional victors of the model: Always Defect and Tit for Tat.

## Methods

### Fungus-growing ant symbiosis

Ants in the tribe Attini engage in an obligate mutualism with basidiomycetous fungi (Lepiotaceae and Pterulaceae) [1,35]. The fungus is maintained in specialized gardens, often subterranean, and ant workers forage for substrate to support the growth of their fungal mutualist and help protect it from potential competitors or parasites. The fungus is vertically transmitted between generations, with new queens carrying a fungal pellet, collected from their natal garden, on the nuptial flight [36]. In exchange for these benefits, the fungus serves as the primary food source for the ant colony. Fungal cultivation in ants has a single origin, ~45 million years ago [37]. The subsequent evolutionary history has generated a diverse collection of ants (more than 230 species) and fungi.

The fungus-growing ant symbiosis is a good model system to investigate the ecological and evolutionary effects parasites have on mutualists for several reasons. First, the symbionts are widely distributed in the new world tropics, and are conspicuous and populous enough to allow for adequate collection. Second, symbionts are amenable to laboratory maintenance, being readily cultivable, thus allowing researchers to study and manipulate each symbiont separately and in combination. Third, an entire tribe of ants culture fungi for food. Each lineage in the tribe tends specific fungal cultivars, which each host specialized mycoparasites in the genus *Escovopsis *(Ascomycota: Hypocreales). *Escovopsis *exploits the ant-fungal mutualism by extracting nutrients from the fungal mycelium at a significant cost to both the ants (indirectly) and fungi (directly) [28,38]. The high prevalence of *Escovopsis *and early origin in the ant-fungal symbiosis [28,31] suggests that it could help stabilize the ant-fungal mutualism over evolutionary time by aligning the selfish interests of the partners against the parasite.

### Experimental design and overview

To empirically test the potential role *Escovopsis *plays in stabilizing cooperation between fungus-growing ants and their cultivated fungi, we investigated the interaction between the fungus garden parasite and cooperative and uncooperative ("cheating") pairs of ants and fungi. The benefits gained by cheating could be diminished if there is a severe parasitic infection which results in increased costs to ants or fungi. In this instance, *Escovopsis *could help stabilize the ant-fungus mutualism, by selecting against cheaters. More specifically, if cheating by either mutualist results in increased morbidity due to the garden parasite, then the selective advantage of cheating would be reduced or potentially nullified. We examine this possibility by using a two-by-two factorial design, crossing the presence/absence of parasitism with the presence/absence of a cheating partner. Sub-colonies were randomly assigned to one of four treatments: i) no infection and no cheating, ii) no infection and cheating, iii) infection and no cheating or, iv) infection and cheating. "Ant-cheating" was simulated by altering the male to female ratio (worker castes are always female, while the only function of males is reproduction) in *Trachymyrmex *cf.* zeteki *sub-colonies. The ant-cheating treatment mimics ant colonies investing more energy into colony reproduction and less into workers that tend the garden. Consequently, there is less investment in colony/garden maintenance (by worker ants), and additional costs imposed on the fungus garden while males inhabit the nest. In separate *Atta colombica *sub-colonies, "fungus-cheating" was simulated by removing the specialized nutrient-rich hyphal swellings (gongylidia) produced by the cultivated fungus for the ants to feed on. Gongylidia benefit ants but they are not necessary for cultivar growth or survival [39], thus removing gongylidia simulates fungal cheating by decreasing the nutrient benefit the fungus provides the ants. Colony fitness following treatment was assessed by measuring fungal garden biomass fluctuations (note: number of ant workers within colonies and colony production of reproductives is highly correlated to fungus garden biomass) [28]. The parasitism treatment involved infecting the ants' fungus garden with the specialized parasite *Escovopsis*. Each treatment is described in detail below.

### Sub-colony setup

Ten six-month-old queenright *A. colombica *colonies and 10 queenright *T*. cf. *zeteki *colonies with a single fungus chamber were collected in Gamboa, Panama. Sub-colonies of *A. colombica *were composed of 1.0 g of fungus garden and ~115 ants (consistent ratio of worker size and age castes, and brood), and were maintained in plastic dual chambers (one housing the garden, and one for feeding, foraging, and dumping of refuse) connected by plastic tubes. *Trachymyrmex *cf. *zeteki *sub-colonies were composed of 0.1 g of fungal cultivar and four ants, and housed in plastic Petri dishes (60 mm diameter). Colonies were placed on mineral oil islands to prevent potential transfer of microbes between sub-colonies via vectors (e.g., mites), were given unrestricted access to foliage (*A. colombica*) or a mixture of dried oats and oak catkins (*T*. cf. *zeteki*), and watered three times a week. All sub-colonies used in the experiment were healthy, stable, incorporating new substrate into the fungus garden, and free of detectable *Escovopsis *infection [15].

### Simulation of cheating by ants

To simulate cheating by the ants the sex ratio of *T*. cf. *zeteki *sub-colonies was altered; in cheater sub-colonies two females and two males were present, while four females and zero males were present in control sub-colonies. We use colonies of *T*. cf. *zeteki *to simulate cheating by the ants because this species regularly produces males in the laboratory (*A. colombica *do not). All worker ants are female, and female reproductives (gynes) are responsible for maternal vertical transmission of the fungal mutualist. Males are reared on the nutrients of the fungal mutualist and stay within the fungus garden prior to the nuptial flight however they do not contribute towards tending the fungus garden. Thus, male ants are a direct cost to the fungus garden; they provide no known benefit to the ant's fungal mutualist, neither dispersing nor contributing to fungus garden maintenance [32].

### Simulation of cheating fungi

To simulate cheating by the fungal mutualist, 10% of *A. colombica *garden biomass (containing ~276 gongylidia clusters) and all gongylidia clusters on the top surface of the garden (~265 clusters per nest) were removed. Gongylidia removal was done by hand, using a dissecting scope (Accu-Scope, Sea Cliff, NY) and jewel-tip forceps (Bioquip, Rancho Dominguez, CA). *Atta colombica *was used to simulate cheating by the fungal mutualist because the cultivated fungus of this species produces large, tightly clustered, nutrient-rich hyphal swellings, called gongylidia. Worker ants preferentially feed on gongylidia and harvest them as nutrients to support the growth of larvae [32,40]. The production of gongylidia by the cultivated fungi provides no apparent benefit to the fungus, but instead serves as a food source that is more beneficial to the ants than the regular hyphae of the fungus [39,41]. Gongylidia do not directly benefit the fungus garden and they do not help defend the garden from *Escovopsis *(see below). While *T*. cf. *zeteki *fungi also produce gongylidia, they are smaller, less abundant and fewer per cluster than those of *A. colombica *(A. Little pers. obs.). To achieve sufficient gongylidia removal and limit fungus garden destruction during sub-colony treatment preparation, it was necessary to use *A. colombica*, rather than *T*. cf.* zeteki *sub-colonies to mimic fungal cheating.

### Infection of sub-colonies with Escovopsis

*Escovopsis *strains used in experiments were isolated in Gamboa, Panama from *T*. cf. *zeteki *and *A. colombica *colonies. Isolates were grown on potato dextrose agar (PDA) (Difco, Sparks, MD) with 1000 iu/ml of penicillin-streptomycin (MP Biomedicals Inc., Aurora, OH). Spores were added to ddH_2_O with Tween 20 [5 × 10^-5^] (Fisher Scientific, Pittsburgh, PA) to evenly disperse spores in solution. *Trachymyrmex *cf. *zeteki *and *A. colombica *colonies received 0.05 and 0.5 ml of solution, respectively (ca 6000 spores/*T*. cf. *zeteki *sub-colony, ca. 20000 spores/*A. colombica *sub-colony) via mist inoculation. Sub-colony biomass was measured prior to, and 72 hours after treatment. The relative changes in biomass/sub-colony/treatment were subjected to 2-way ANOVA in Minitab [42]. The success of the ants is directly dependent upon the health and biomass of the fungus gardens, therefore, as in other studies [43,44], we use garden biomass as an indirect fitness indicator for the ant in the ant-cheating experiment.

### Prisoner's Dilemma computer simulation

Using the classic Prisoner's Dilemma model (PD) [34], we further explore our empirical results that indicate parasites can play a role in stabilizing cooperation. In the PD two players interact, each has the ability to cooperate or cheat. Cooperation provides the opponent a benefit (*b*), while incurring a cost (*c*) to the player (b > c > 0). The highest payoff is received when a player cheats while its partner cooperates: the cheater benefits without paying the cost of cooperation (*T*emptation to cheat *T *= *b*). If both players cooperate each receives a net benefit (*R*eward) of *R *= *b *- *c*, while mutual cheating results in a *P*unishment payoff of *P *= 0. The lowest payoff is received by a player that cooperates while its opponent cheats (*S*uckers payoff *S *= *-c*). In single interactions, cheating is the best strategy (*T *> *R *> *P *> S). Our results from ant-fungal manipulations suggest that the presence of a parasite would alter the PD payoff matrix such that pure cooperation is the best strategy. More specifically, cheating by either ants or fungi results in an increased parasite impact, reducing the benefit of 'temptation' to cheat (*R > T = P > S*). In addition, because cooperation by one player (i.e. ants) provides some degree of defense against parasitism (i.e. *Escovopsis*) further alters the payoff matrix to favor cooperation (R > T = P = S).

The Iterative Prisoner's Dilemma Model (IPD), where players engage in multiple interactions, is much more relevant to natural system. Based on the alteration of the payoff matrix of the single interaction PD model (see above), it is clear that if parasites impact every interaction in the IPD model they will help favor cooperation over cheating. However, it is very unlikely that parasites are so ubiquitous in natural populations that they influence every interaction. Thus, we utilized a computer program called DILEMMA to determine what level of parasite prevalence is required to potentially help stabilize cooperation within the IPD model [see Additional file 1].

Using DILEMMA, we explored the role of parasites in altering the dynamics within the IPD model by conducting simulations involving various combinations of strategies in the presence and absence of parasites. Simulations involved populations of 10,000 individuals, each individual engaged in 25 interactions per generation. Simulations were run for 500 generations, which we previously determined to be sufficient to obtain a stable proportion of strategies across generations. The average of 100 independent runs for each different simulation is presented. In the first simulation a 50:50 ratio of individuals playing 'always defect' (uncooperative strategy), and 'always cooperate', (cooperation in every interaction) was used. Subsequently, simulations using the same 50:50 ratio with parasites present were run. Ten independent runs (500 generations each) were run with proportions of parasitism increasing by increments of 10%, and the means of the final frequencies of each strategy in the population were plotted by proportion of population infected with parasites. A second set of simulations was conducted, with three additional strategies ('tit for tat', 'sneaker', and 'random' [see Additional file 1]). All strategies started with a 20% frequency in the population. As above, this simulation was run in the absence of parasites for 500 generations, and then parasites were added with varying prevalence up to infection rates of 100%.

## Results

### Empirical test of hypothesis

**Figure 1:**
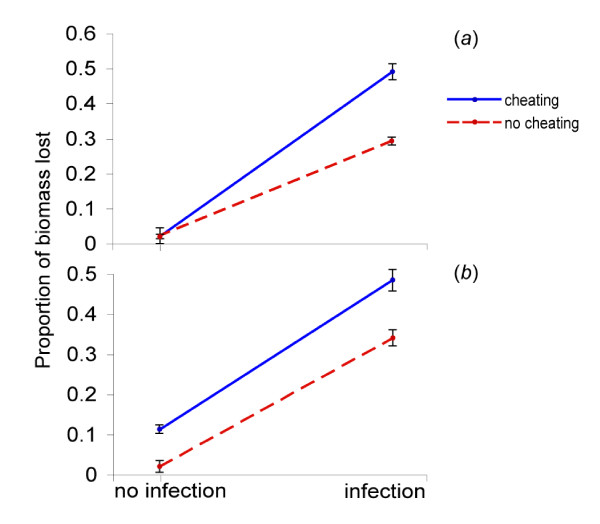
**Cheating/infection experiments**. Two-way interaction graph illustrating the impact of experimental infection and cheating ants or mutualistic fungi on the garden biomass in two types of fungus-growing ant colonies. **A) ***Trachymyrmex *cf.*zeteki *sub-colonies (n = 40), treated with crossing the presence/absence of fungus garden infection with the presence/absence of cheating ants, shows significantly higher loss of garden biomass when exposed to cheating and infection simultaneously than in all other treatments (2-way ANOVA p < 0.001 df = 3). **B) ***Atta colombica *sub-colonies (n = 80), crossing the presence absence of fungus garden infection with the presence/absence of cheating fungi, shows a significantly greater loss in garden biomass when cheating and infection are experienced simultaneously than in all other treatments (2-way ANOVA p < 0.001 df = 3). Non-parallel lines shown in '**A**' and '**B**' illustrate significance (2-way ANOVA analyses) of the interaction between infection and cheating treatments.

In our experiments we found that infected sub-colonies with cheating populations of ants or fungi each lost significantly more garden biomass than sub-colonies subjected to infection or cheating (ants or fungi) alone (2-way ANOVA p < 0.001 df = 3, for both treatments) (Fig. 1). When a cheater is present, the cost of *Escovopsis *infection is substantially greater than it is in sub-colonies with only cooperative partners. This suggests that the negative consequences *Escovopsis *has on ant and fungal health could result in parasite-induced selection eliminating cheating by either mutualist. There are several reasons colonies with cheaters are likely to be less successful at fighting garden infection, than those with cooperative ants and fungi. Increased virulence of *Escovopsis *in the "cheating ant" treatment is likely because the enforced shift to a 50% male:worker ratio results in fewer worker ants present to defend the garden from infection [44]. In the "cheating-fungi" treatment, the mechanism(s) causing a greater impact of infection is less clear. The high concentration of nutrients found in gongylidia may be a necessary energy source for worker ants that remove parasitic spores. The ants may also retaliate against fungal cheaters by allocating less effort into garden maintenance, which would result in greater garden biomass loss during infection.

### Prisoner's Dilemma simulations

**Figure 2:**
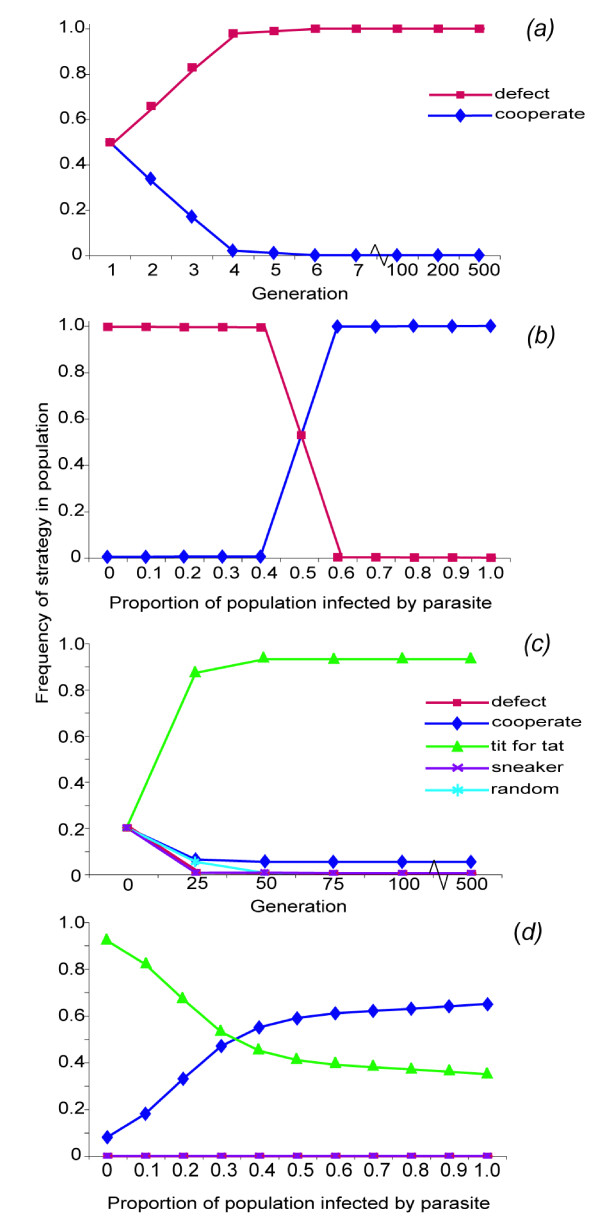
**Prisoner's Dilemma simulations**. Graphical output from DILEMMA, a computer program simulating the Iterated Prisoner's Dilemma (IPD) with the ability to incorporate parasites into the classic model to determine how prevalent parasite would need to be within populations to help favour cooperation over cheating. **A) **In the traditional IPD, the strategy "always defect" quickly eliminates the purely cooperative strategy "always cooperate" from the population. **B) **When parasitism is introduced into the population, "always cooperate" becomes a dominant strategy when infection rates are greater than 50% in the population. **C) **Strategies that combine cooperation with retaliation, such as "tit for tat", are successful in a heterogeneous population of strategies in the IPD (strategies defined in Supplementary Table 2). **D) **In the presence of parasites, "always cooperate" becomes a viable and dominant strategy in a heterogeneous population at parasitism levels as low as 35%. Output was generated using populations of 10,000 individuals, which engaged in 25 interactions per generation, for 500 generations. Each graph depicts the mean of 100 independent runs of the DILEMMA program. Standard error (SE) bars are not shown because values are less than 4.92 × 10^-3^, with the exception of the data points at 0.5 parasitism *(b)*, which have SEs of 0.479.

In the classic model, when one partner always cooperates and the other always cheats, the cheater population quickly out-competes the cooperator population (Fig. 2a). In contrast, parasite infection rates of 51% or higher results in the strategy 'always cooperate' being successful and stable (Fig. 2b). In the classic model cooperation is successful and stable if cooperative strategies can retaliate against cheating, such as the well-known IPD strategy 'tit for tat' (TFT, Fig. 2c). In our DILEMMA simulations, when cooperative strategies capable of retaliation against cheaters (i.e., TFT) are included, the strategy "always cooperate" forms a stable population at infection levels of 10%, and out-competes TFT when infection levels are greater than 35% (Fig. 2d). These simulations support our empirical results by indicating that when parasites are common, cooperation is stable as the benefits gained by cheating are outweighed by the increased cost of infection.

## Discussion

Despite the important role mutually beneficial associations play in shaping all levels of biological organization, how these relationships establish and maintain stability is not well understood. The challenge is elucidating the factor(s) that prevent selection from favoring partners who pursue their own selfish interests, cheaters who obtain benefits without providing rewards in return. Most theories proposed to help explain the evolutionary stability of mutualism argue that cooperation is stabilized by individuals employing mechanisms to avoid being exploited by their partners (e.g., host sanctions, partner choice) [8,45]. In contrast to this typical view that partners enforce reciprocity within beneficial exchanges, here we suggest, and provide empirical and theoretic evidence for, the possibility that an external force, such as parasitism, can help stabilize cooperation by aligning the selfish interests of partners.

**Figure 3:**
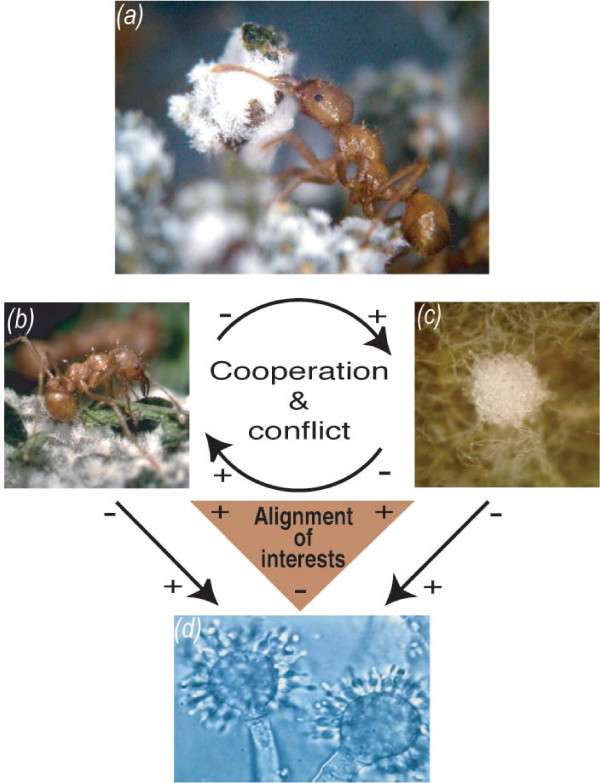
**Cooperation and conflict within the fungus-growing ant microbe symbiosis**. **A) **Fungus-growing ants forage for substrate to nourish their cultivated fungus, which they also groom to help remove garden parasites. **B) **In return, the fungus serves as the primary food source for the ants; with some species producing nutrient-rich hyphal swellings (gongylidia) that the ants preferentially feed on. Cooperation and conflict is inherent to the ant-fungus mutualism (black arrows, head points toward recipient of benefit), with each symbiont receiving a benefit (+), at a cost to the other (-). Natural selection favors symbionts that increase their own fitness selfishly by exploiting their partner, receiving a benefit (+) without paying the cost (-) associated with providing a benefit in return. **C) **The mutualism is parasitized by specialized fungi in the genus *Escovopsis*, which acquire nutrients from the fungus garden at a direct and indirect cost to the cultivated fungus and ants, respectively. Cooperation is enforced, and cheaters minimized, because the selfish interests of both ants and cultivated fungus are aligned (orange triangle) against the parasite *Escovopsis*.

One way parasites may help stabilize mutualisms is if 'cheating' by one partner results in greater parasite-induced morbidity or mortality in one or both partners, resulting in a net loss to the 'cheater' (i.e., the benefits obtained from 'cheating' are diminished by the increased costs from more severe infection by the parasite). Indeed, here we found, using the fungus-growing ant mutualism as a model system, that cheaters can suffer disproportionately more in the presence of a parasite than their non-cheater counterparts. More specifically, enforced cheating by either the ants or their fungal partner had little to no negative impact on the health of the fungus garden, which both mutualists obligately depend on. In the presence of the garden parasite, cheating by either mutualist resulted in significantly higher parasite induced garden morbidity, as compared to controls involving garden infections with cooperative mutualist partners. Thus, our empirical results indicate that the increased impact of parasitism in the presence of cheating can reduce the inherent conflict [46-48] between mutualists (Fig. 3), potentially contributing to the stability of the beneficial association.

*Escovopsis *can be extremely prevalent, infecting more than 75% of colonies of fungus-growing ant nests in some populations, and is known to be virulent [15,28]. Thus, we believe our results indicate that cheating by either ants or fungi could be rapidly eliminated within natural populations by previously established infections or by new infections of the horizontally transmitted parasite. The alignment of interests between the ants and their cultivated fungi, in opposition to the garden parasite, is further illustrated by the contribution the ants make to cultivar defense. Specifically, the ants employ specialized behaviors to physically remove parasitic inoculum from the fungus garden [44]. Without ant behavioral defenses, the garden is rapidly overgrown by the parasite [15], indicating that defense against *Escovopsis *requires cooperation between ants and their fungal mutualists. The early origin of *Escovopsis *within the symbiosis and its coevolutionary history with the ants and their fungal cultivar [31], suggests that the parasite may have been a stabilizing force within the ant-fungal mutualism for millions of years.

Our view of the stability of cooperation has largely developed out of game theory, especially the PD model [34]. In the classic single interaction model, cheating is always favored over cooperation (*T *> *R *> *P *> S, see methods above and Fig. 2a). However, when the model involves multiple interactions among players (IPD), strategies that are cooperative but capable of retaliating against cheating can out-compete cheating strategies (e.g., the well-known TFT). As outlined above, our empirical results indicate that a parasite has the potential to alter the payoff matrix so that cooperation is favored over cheating in the single interaction PD model. This illustrates the potential for third parties to alter the dynamics of cooperation in ways that shape mutualism stability. Our simulations revealed that even at relatively low prevalence parasitism can select for stability of a cooperative strategy that is incapable of retaliating against cheating. Specifically, 'always cooperative' out-competes 'always defect' when 51% of interacting partners in a population are infected, which is well within the known infection rates in the fungus-growing ant mutualism. When TFT was integrated into the simulation, surprisingly, we found that 'always cooperative' forms a stable population at infection levels of 10%, and out-competes TFT when parasite prevalence is greater than 35%. These findings provide theoretical support to our empirical results from the fungus-growing ant mutualism, further suggesting that parasites can provide an external sanction against one partner's cheating, or simply alter the costs and benefits received from cooperation versus cheating in such a way that natural selection favors cooperation.

We believe our findings are applicable beyond the fungus-growing ant microbe-symbiosis. Mutualisms in which survival and reproduction are tightly linked to cooperation are especially likely to be stabilized by antagonists, as morbidity and mortality in one partner is expected to have a significant cost to the other; this is complimentary to partner fidelity feedback [8]. Protective mutualisms, in which one partner defends the other from a natural enemy, are common in nature. Just as fungus-growing ants protect their mutualistic fungi from parasites, there are ants that protect plants from herbivores [49], bacteria that protect their insect hosts from disease [29], and endophytic fungi that protect their plant hosts from herbivores via secondary metabolite production [50]. Our results support the prediction that in these interactions it is likely that when the threat imposed by a tertiary symbiont (i.e. predator, parasite) is absent, the protective mutualism may break down. Indeed, a recent paper by Palmer et al. [51] revealed the breakdown of an ant-plant mutualism in the absence of large-herbivores. Furthermore, the contribution symbionts make to protect their hosts, which appear to be widespread (see [52]) may be evidence of parasites aligning the interests of mutualists.

## Conclusion

Cooperative relationships that occur in natural systems persist in complex ecological communities where interspecific interactions are continuous. In some instances one, or a combination, of the models included in the Sachs et al. framework of directed reciprocation, shared genes, and by-product benefits, adequately explains stable cooperation among organisms. However, our results suggest that a third species eliciting selective pressure on one member of a mutualism can limit cheating by a mechanism that does not neatly fit the current framework. Cooperative dynamics in which two partners have their selfish interests aligned in opposition to a third (parasitic) party, can provide a stabilizing force that helps maintain cooperation between species, that is neither a by-product (e.g. coincident of a selfish action), nor directed reciprocation. Additionally, it is important to be clear that parasitism need not be a mutually exclusive factor stabilizing cooperation. It is possible, and perhaps likely, that parasite pressure works in concert with other well-defined mechanisms that promote cooperation. It would be interesting to empirically test how the addition of a third parasitic species influences cooperative interactions that are believed to be governed by reciprocation, by-product benefits or shared genes.

## Authors' contributions

AL and CC conceived and designed the experiments. AL performed the experiments and analyzed the data. AL and CC wrote the paper and approved the final manuscript.

## Supplementary Material

Additional file 1 **Dilemma information** Click here for file
