# Supplementary material for: Parasites may help stabilize cooperative relationships
Source: BMC Evol Biol. 2009 Jun 1;9:124. doi: 10.1186/1471-2148-9-124 (PMC2701933; doi:10.1186/1471-2148-9-124)

The DILEMMA program is an IPD simulation that incorporates parasitism (the program is available at <http://www.bact.wisc.edu/currie/dilemma>).

**Dilemma program**

The interface for the DILEMMA program is divided into four sections (Fig. S2). In the box titled ‘Parameters’, the user sets the population size (an even number greater than or equal to the number of strategies), the number of rounds which are the interactions between partners per generation, the number of generations per iteration, and the number of iterations, which is the number of sets of generations in the run.

In the ‘Payoffs’ box are two matrices. The Regular matrix contains the classic values used in the IPD. This is the matrix used when partners interact without parasitism. The Parasitic matrix, lower, contains the payoff scores received by each partner when they experience parasitism; default values are 3,0,0,0, the values used in the manuscript. The values in both matrices can be altered by the user.

In the ‘Parasitism’ box, the user first selects whether parasitism is fixed or varied. If parasitism is fixed, all iterations completed will be at that fixed level of parasitism (0.0–1.0). If parasitism is varied, iterations will be completed at each level of parasitism the user chooses (0.0–1.0 by some interval between 0.001–0.5). In the first case, results will be displayed on a graph with strategy frequency on the Y axis, and generation number on the X, while if parasitism is varied, the X axis will instead report parasitism levels by the chosen interval. In the latter case, output is the mean frequency of the strategies in the population over x generations and y iterations**.**

In the ‘Strategies’ box strategies are selected or deselected by the user. Strategy frequencies, assigned to the population of prisoners in the first generation, may be set by the user (totalling 1.0), or automatically calculated and set to equal values. There are ten strategies to choose from (Table S1). The lower half of box four allows the user to select one of four types of parasitism to inflict on partners (Table S2).

**Justification for payoff matrix**

In our modified IPD simulations we used the payoff matrix of R = 3, S = 0, T = 0, P = 0 (Table S1), which we believe accurately reflects the cost and benefits of interactions within the fungus-growing ant symbiosis. The garden parasite *Escovopsis* is a virulent parasite that can rapidly and completely overwhelm whole intact fungus gardens if not controlled (Currie et al. 1999a, Currie 2001). Even if controlled to sub-lethal levels the parasite can establish persistent infections that dramatically reduce the accumulation of the fungus garden (Currie 2001). This decrease in growth rate could reduce colony fitness to zero, as the ant colonies must reach a significant size to produce new reproductive individuals. Since the cultivated fungus is vertically transmitted, by new queens carrying the fungus from parent to offspring nest, both lethal and virulent persistent infections would also reduce the fitness of the mutualistic fungus. Thus, our payoff matrix reflects the following: i) if the ants and the mutualistic fungus do not cooperate the increased virulence of *Escovopsis* results in a cost that reduces any benefits received from non-cooperation (P = 0), and ii) in the absence of cooperation the impact of the parasite can even reduce the payoff normally received from the temptation to cheat (T = 0).

**Test to determine whether gongylidia protect the fungus garden from *Escovopsis***

We experimentally tested the possibility that removing gongylidia has a direct impact on the fungus gardens’ ability to defend itself from *Escovopsis* infection. Specifically, we removed ten, 0.1 g pieces of garden from each of the four *A. colombica* colonies used in treatments. All gongylidia clusters were removed from five, randomly chosen, garden pieces from each colony. Garden pieces were then placed in Petri dishes (60 mm diameter) along side a 4 mm diameter plug of *Escovopsis* grown on PDA. Growth of *Escovopsis* was monitored and scored 36 and 48 hours after exposure to garden pieces. After 36 hours there was no significant difference in *Escovopsis* growth in the two treatments; 80% of the control garden pieces were overgrown by *Escovopsis*, and 85% of garden pieces with gongylidia removed were overgrown (G = 0.174, d.f. = 1, p > 0.1). After 48 hours, all garden pieces were overgrown by *Escovopsis*.

**Table S1.** Description of the different strategies available for use in the DILEMMA program.

| Strategy name | Description of the strategy |
| --- | --- |
| Always Cooperate | A partner assigned this strategy will always cooperate (C) |
| Always Defect | A partner assigned this strategy will always defect (D) |
| Alternate | Cooperate in the first round of each generation, and alternate (D,C,D,C etc.) thereafter |
| Grudger | Cooperate in the first round of each generation. Cooperation continues until partner defects, then always defect (for the rest of that generation) |
| Random | Cooperate or defect with equal probability and without regard to partner’s strategy |
| Tit for Tat | Cooperate in the first round of each generation, then act as one’s partner did in the previous round |
| Tit for 2 Tats | Cooperate in the first and second round of each generation; after that if one’s partner defected in each of the previous two rounds then defect, otherwise, cooperate |
| Big Memory | Cooperate or defect with equal probability in the first round. Then cooperate with probability “p” where p = (partner’s C count) / (partner’s C count + partner’s D count) |
| Sneaker | Cooperate in the first round of each generation. Thereafter, act as your partner did in the previous round, but additionally defect with user-set probability “p” |
| Pavlov | Cooperate in the first round of each generation. Thereafter, if one’s last round score was equal to the Regular Payoff CC or the Regular Payoff DC score, then repeat your previous act, if not, switch actions |

**Table S2**. Description of how the different types of parasitism are imposed on partners in the DILEMMA program. The user selects the type of parasitism, as well as the prevalence of parasites.

| Parasitism type | Description of parasitism type |
| --- | --- |
| Simple | Parasitism (i.e., applying the parasitic payoff matrix to infected pairs in place of the regular payoff matrix) is randomly assigned to pairs before each round, but does not persist beyond that round. |
| Severe | Parasitism is randomly assigned to pairs before every round, and is persistent; a parasitized pair remains parasitized for the remainder of that generation. |
| 1st-Round | Parasitism is randomly assigned to pairs before *the first* round of each generation, and persists through all subsequent rounds in the generation. No additional parasitism is introduced in subsequent rounds of that generation. |
| Weak | Parasitism is randomly assigned to pairs before the first round of each generation. The round in which parasitism first appears is randomly assigned to each parasitized pair. Parasitism is persistent through subsequent rounds once it first appears. Additionally, the round in which parasitism initially appears may be Uniformly or Normally distributed across the number of rounds, as set by the user. |

**Figure S1.** Payoff matrices used in our simulations to explore the potential role of parasites stabilizing mutualisms. (*a*) The default matrix used for the classic Prisoner’s Dilemma Game , after Axelrod & Hamilton (1981). (*b*) Default matrix used to distribute scores among players when infected by a parasite.


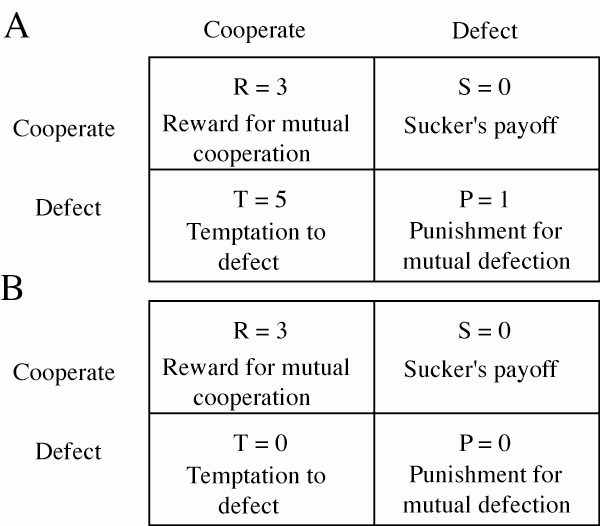


**Figure S2**. User interface of the DILEMMA program designed to incorporate parasitism into the classic Iterated Prisoner’s Dilemma game.


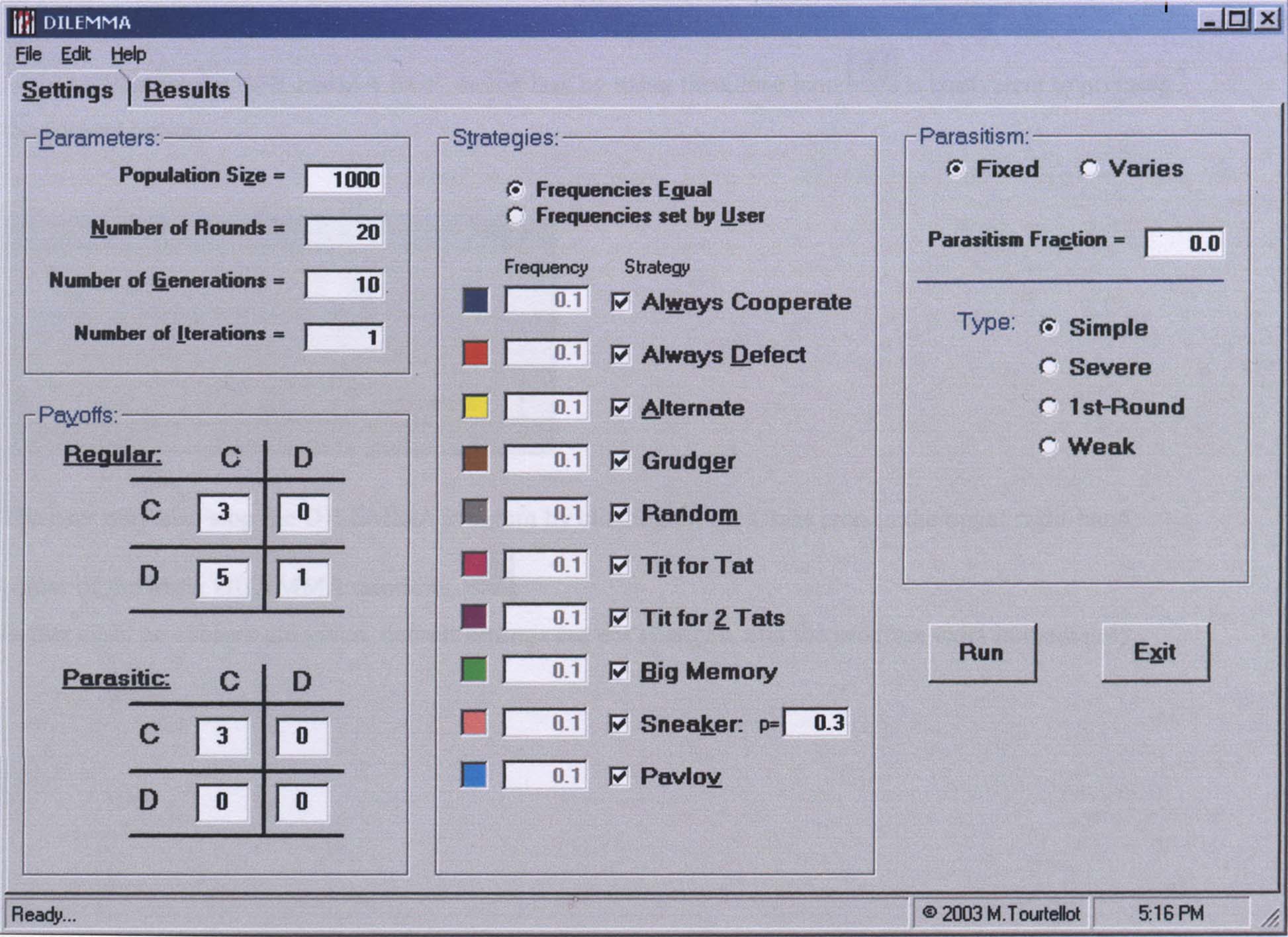

Supplement: Additional file 1 — Dilemma information [file 1471-2148-9-124-S1.doc]
